# Assessment of Knowledge and Attitude of Medical Students Towards Nutrition and Health: A Cross-Sectional Study

**DOI:** 10.7759/cureus.68329

**Published:** 2024-08-31

**Authors:** Siddhika Kesapragada, Anita Teli, Lata Mullur

**Affiliations:** 1 Physiology, Shri B.M.Patil Medical College, Hospital and Research Center, BLDE (Deemed to be University), Vijayapura, IND

**Keywords:** nutrition - related diseases, diet, medical students, health, nutrition

## Abstract

Introduction: Students' health and well-being generally depend on their nutritional intake. Medical students, in particular, must be physically and mentally fit to perform better academically and to treat their patients holistically. Consuming a nutritious diet and maintaining good health requires an adequate understanding of nutrition and related diseases. Thus, this study used a validated general nutrition knowledge questionnaire to evaluate nutritional knowledge and aimed to assess medical students’ knowledge and attitudes about health and nutrition-related diseases.

Methodology: A cross-sectional study was conducted on 123 medical students between July 2023 and June 2024. A general nutrition knowledge questionnaire was used for the online survey, which consisted of questions on nutrition knowledge, individual dietary choices, thoughts about expert advice and awareness about health problems and diseases related to weight management and diet. The responses obtained were put in an Excel (Microsoft, Redmond, WA, USA) spreadsheet, and the data obtained was transformed into bar graphs and pie charts by using SPSS version 20 (IBM Corp., Armonk, NY, USA). For the statistical analysis of data, percentages were calculated.

Results: The mean age of the study participants was 18.56 ± 1.16 years. The gender-wise distribution of participants shows around 47.7% (50) male and 57.3% (66) female. 39.8% of students answered correctly the precise requirement of macronutrients. 21.1% of students were unsure about the content of added sugar, salt and fibre requirements. The majority of students made healthy dietary choices, while many students were unaware of the expert’s advice and diet-related diseases.

Conclusion: It was discovered in the current study that students’ general understanding of a nutritious diet was good, but they lacked knowledge regarding the nutrient content. Students showed a sufficient level of understanding of the healthy food selections, but they were not aware of the healthy cooking methods. Students were unaware of the advice given to the patients, while awareness of nutrition-related diseases was acceptable. Inadequate understanding and lack of awareness are attributed to the limited nutrition-related curriculum in medical schools.

## Introduction

Adolescent health and well-being are greatly influenced by nutritional intake, which is something that matters more to them [1]. A student’s health is the most critical aspect of their life, largely dependent on their food type. It directly or indirectly affects the student’s mental, physical, and cognitive growth [2].

Moving to college can be difficult for many young people, marked by growing independence, socialising, self-control, and self-organisation [3]. Medical students, in particular, are more likely affected by exhaustive schedules and humongous syllabi that force them to be seated. Studying for long hours often leads to snacking on unhealthy, packaged foods that are easily accessible and seem to be convenient for the majority, but what they fail to understand are the long-term effects of doing so. Poor health often interferes with daily living and can hinder a student’s learning. A healthy student is likelier to perform better academically and be more social and active. Unhealthy food intake patterns are instead associated with significant long-term consequences, including cardiovascular diseases, asthma, clinical depression, stroke, and cancer in later life. Thus, students’ food intake quality has become a significant concern [4]. As medical students, it is essential to be rightly educated about nutrition and health so that, as practising physicians, they can advise and guide patients towards a healthier life. Nutrition knowledge needs to be taught better in medical schools, which will help students learn and apply themselves to better academic performance.

Results from previous cross-sectional studies on college students indicate a need for more understanding of various dietary themes. Specifically, students incorrectly answered more than 50% of the questions about fruit and vegetables, milk, its substitutes, and fermented dairy products [5], vitamin D [6], food labels [7], and the connection between diet and chronic illness [8,9].

A validated instrument for evaluating nutrition knowledge in adults, the General Nutrition Knowledge Questionnaire (GNKQ), was created by Parmenter and Wardle (1999) [10] in the UK. It has also been utilised in college student investigations [8,11]. Research conducted on university students using the GNKQ tool to measure knowledge revealed that the mean scores of correct answers varied from 51% to 67%, indicating a modest degree of general knowledge [9,12].

Comprehending the present state of students’ knowledge aids in creating focused and more effective interventions in academic environments [13]. Thus, this study aimed to identify students with poor and good nutrition knowledge and their attitudes towards health-related diseases among the sample of university students in North Karnataka. To our knowledge, no such study is being conducted among medical students using GNKQ in North Karnataka.

## Materials and methods

This cross-sectional study was conducted between July 2023 and June 2024 among medical students of all phases studying in BLDE (Deemed to be University), Shri B.M. Patil Medical College, Vijayapura, Karnataka, India.

The sample size was calculated based on an article by Belogianni et al. [13], with the anticipated proportion of good knowledge of 46.8%; the study would require a sample size of 196 students with a 95% level of confidence and 7% absolute precision, by using n = Z^2^p*q/d^2^, where Z = Z statistic at α level of significance (1.96), d2 = absolute error (7%), p = proportion rate (46.8%), q = 100-p.

Consequently, the study included randomly 200 students who were older than 18 years old and willing to participate. Following the exclusion of the incomplete responses from the study, 123 complete responses were acquired, guaranteeing the highest data quality standards.

The online survey link was sent through the university's email systems and was accessible from July 2023 - June 2024. Participation in the survey was entirely voluntary, it was anonymous, and there were no mandatory questions. Before beginning the study, participants clicked the “agree” button to provide informed consent. Ethical clearance (BLDE(DU)/IEC/1015 - A/2023-24 dated 26/6/2023) for this research project was obtained from the institutional ethical committee of Shri B.M. Patil Medical College, BLDE University, Vijayapura, following a rigorous review process. Ethical clearance was proceeded by permission from the department to conduct the study on medical students. Students willing to participate were briefed about the questionnaire and the need for the study. To the best of our abilities, we ensured participation was fair and voluntary. 

Data collection

A questionnaire was prepared using a Google Form based on the General Nutrition Knowledge Questionnaire, UK [10], a widely accepted and validated tool developed by Parmenter and Wardle in the 1990s. The scientific community widely uses this questionnaire for research purposes due to its comprehensive coverage of nutrition knowledge. The questionnaire consisted of four sections; initial questions were on general and personal information, followed by four sections, where section I was related to nutrition knowledge, section 2 was on questions about individual dietary choices, section 3 was thoughts about expert advice, section 4 was awareness about health problems and diseases related to weight management and diet. The online survey link was sent through the university email systems after a briefing about the study for those who intended to participate. We made sure to maintain accurate participation and ensured the confidentiality of responses. The incomplete reactions were excluded.

Statistical analysis

The responses obtained were put in a Microsoft Excel (Redmond, WA, USA) spreadsheet, and the data obtained was transformed into bar graphs and pie charts using SPSS version 20 (IBM Corp., Armonk, NY, USA). For the statistical analysis of data, percentages were calculated. Post-data collection showed that most students were first-year, followed by second-year and third-year medical students.

## Results

A total of 123 participants were recruited for the study after considering the inclusion and exclusion criteria. The mean age of the study participants was 18.56 ± 1.16 years. The gender-wise distribution of participants shows around 47.7% (50) male and 57.3% (66) female. Out of 123 students, about 70.7% (87) were from Phase I, 13% (16) from Phase II, 6.5% (8) from Phase III Part 1, and 9.8% (12) from Phase III Part 2 in the study. Most students were non-vegetarians (62.6%, n = 77) by diet, followed by lactovegetarian and lacto-ovo-vegetarian at 20.3% (25) each, vegan at 15.4% (19), and pescatarian at 2.4% (3). When asked to rate their health, 8.1% (61) perceived their health as ‘excellent’, 23.6% (29) as ‘very good’, 49.6% (61) as ‘good’, 17.9% (22) as ‘fair’ and only 4.1% (5) as ‘poor’.

There were four sections altogether in the questionnaire. Questions about the students’ dietary knowledge were included in Section I. Section I contained 19 questions in total.

Section I: questions related to knowledge about nutrition

**Figure 1:**
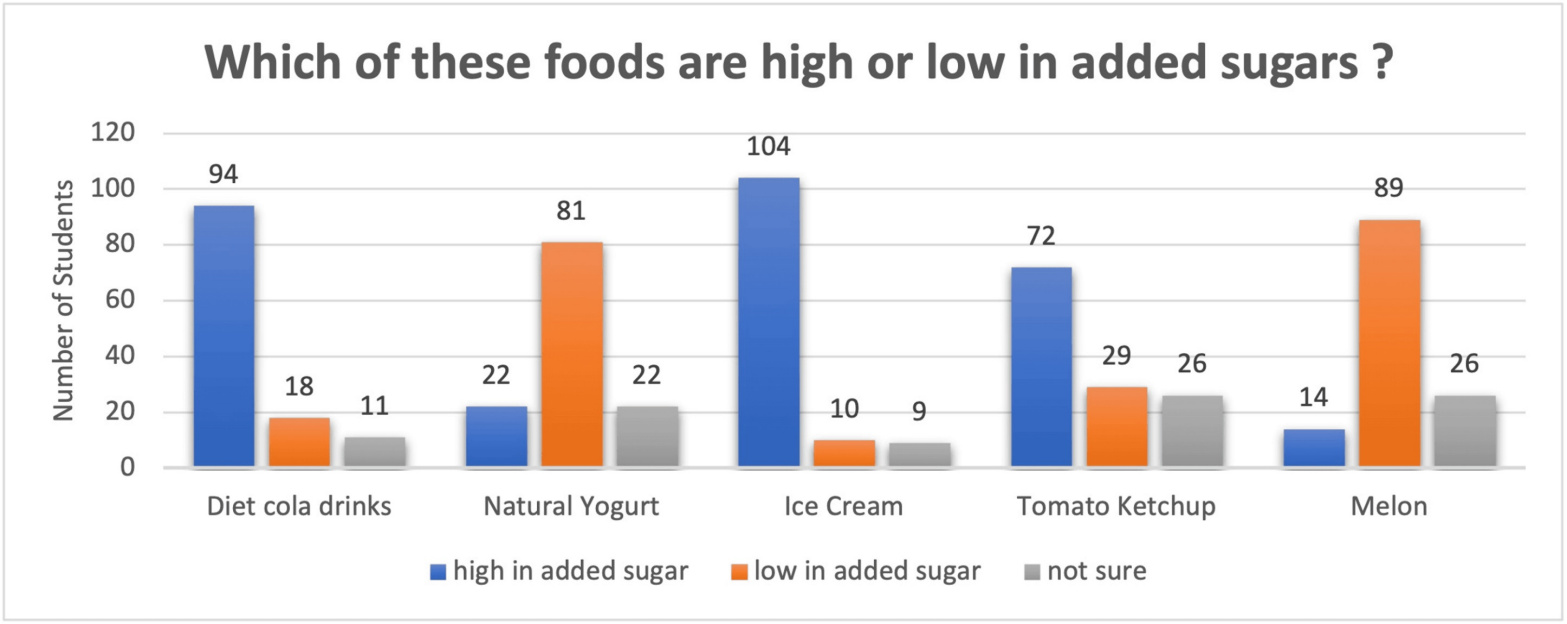
Depicting the knowledge of students about high- and low-added-sugar foods

Which item has high or low added sugar? This was the first question. The response, as seen in Figure 1, showed that 94 (76.4%) identified diet cola drinks had high added sugar, 18 (14.8%) said diet cola drinks had low added sugars, and 11 (8.4%) said they were not sure. Similarly, 81 (65.8%) said natural yoghurt is low in added sugar, 22 (17.8%) said high in added sugar, and 22 (17.8%) were unsure. Ice cream was identified as high in added sugar by 104 (84.5%), low in added sugar by 10 (8.1%) and uncertain by nine (7.3%). Seventy-two (58.5%) thought tomato ketchup was high in added sugar, 29 (23.5%) thought it was low in added sugar, and 26 (21.1%) were unsure. Finally, 89 (72.5%) students said melon is low in added sugar, 14 (11.3%) said melon is high in added sugar, and 26 (21.1%) are not sure about the sugar content of melon.

**Figure 2:**
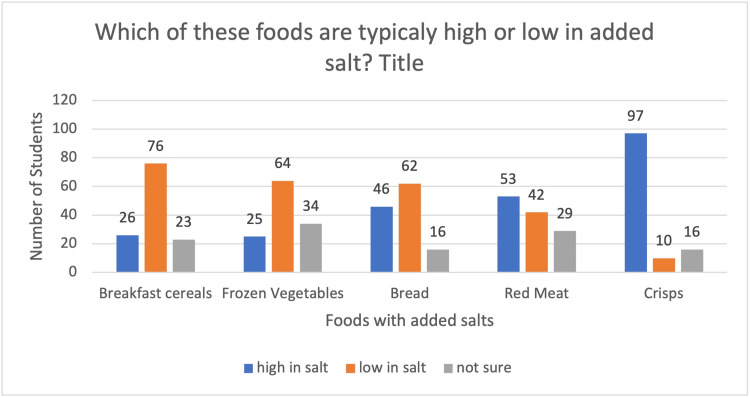
Depicting the knowledge of students about high- or low-added-salt foods

The second query in the section was about high- and low-added-salt foods. A total of 97 (78.8%) pupils said that crisps had more added salt, 53 (43%) said red meat, 46 (37.3%) said bread, 25 (20.3%) said vegetables and 26 (21.1%) indicated that morning cereals have a lot of added salt. Similarly, 76 (61.7%) respondents indicated that morning cereals are low in added salt, 42 (34.1%) showed meat, 62 (50.4%) stated bread, 64 (52%) said frozen veggies and 10 (8.1%) responded that crisps have low added salt. The responses are seen in Figure 2.

**Figure 3:**
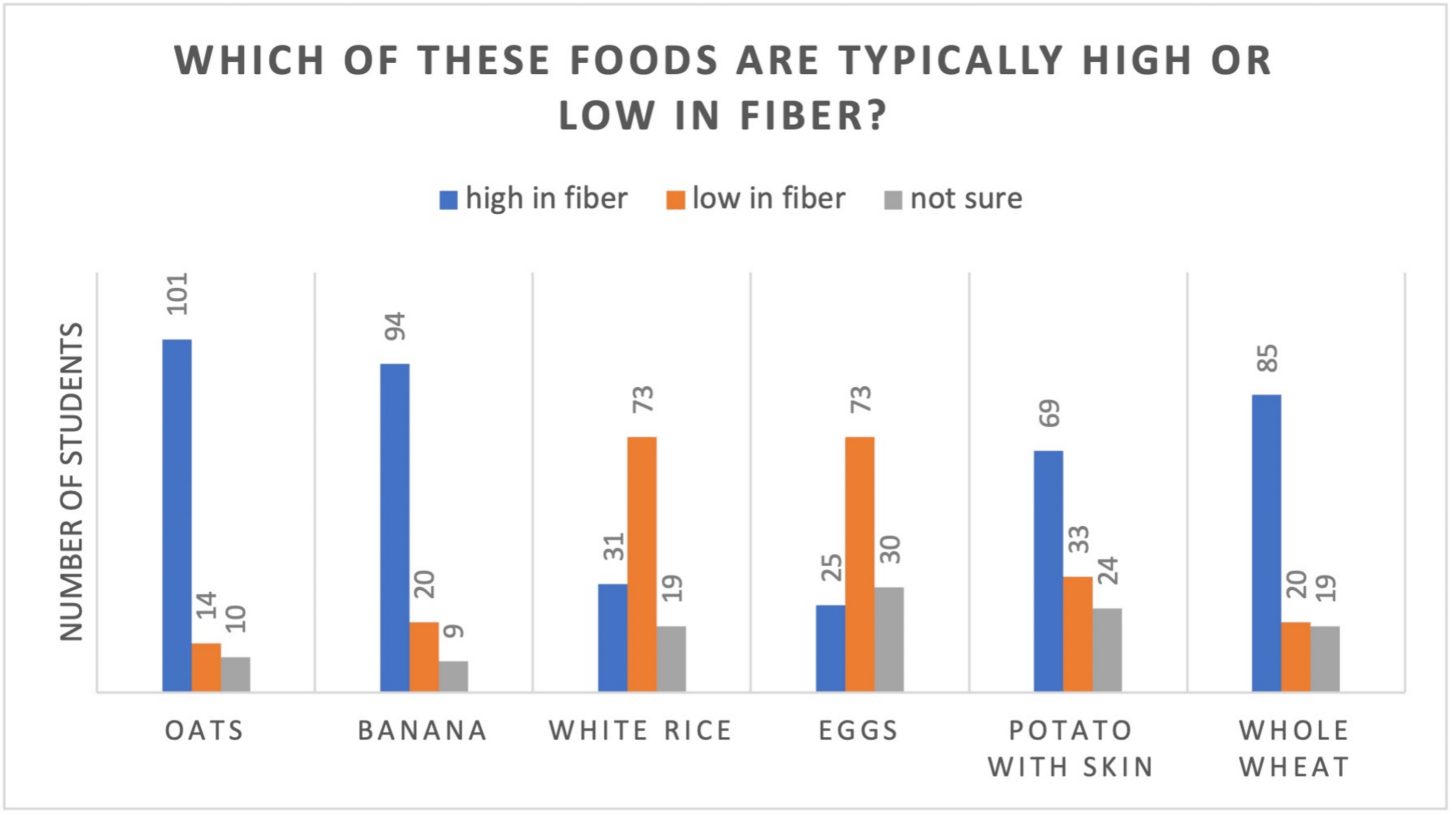
Depicting the knowledge of students about high- or low-fibre foods

In addition, items such as whole wheat, bananas, white rice, eggs, potatoes with skin, and oats were evaluated, and their fibre content was asked to be rated, as depicted in Figure 3. 82.1% of students said oats in high in fibre, 11.3% are low and 8.13% were not sure. Ninety-four (76.4%) said bananas were high in fibre, 20 (16.2%) said bananas were low in fibre, and nine (7.3%) weren’t sure of the fibre content of bananas. Seventy-three (59.3%) answered that white rice is low in fibre, 31 (25.2%) said it is low, and 19 (15.4%) were not sure of the fibre content of white rice. Seventy-three (59.3%) said eggs are low, 25 (20.3%) said high and 30 (24.3%) were not sure of the egg’s fibre content. Thirty-three (26%) said potato with skin is low in fibre, 69 (56%) said high and 24 (19.5%) said they were not sure. Twenty (16.2%) said whole wheat is low in fibre, 85 (69.1%) said it is high, and 19 (15.4%) were not sure of the fibre content of the whole wheat.

Various foods containing protein were included in the subsequent questions, and the responses recorded are depicted in Table 1. The next question was asked to determine the amount of starch in various foods like cheese, potatoes, pasta, almonds, and plantains; responses were gathered and are shown in Table 2. Polyunsaturated fat was identified as the predominant type of fat in olive oil by 36.5%, in butter by 37.6%, in sunflower by 42.2% and in eggs by 35.7% of students, as displayed in Table 3. The highest amount of trans-fat was identified in biscuits, cakes and pastries by 75.6% of students and the lowest in eggs by 5.7%, as shown in Table 4. The calcium content of whole milk compared to skimmed milk was identified as much higher by 37.4% of students, and 18.7% were unsure of the calcium content, as shown in Table 5. Table 6 shows the highest calorie content per weight of the below nutrients. 41.5% of students identified fat as the highest calorie content, and 13% of students said fibre as the lowest calorie content. The calorie content of processed foods compared to minimally processed foods is shown in Table 7.

**Table 1:** Depicts responses of students for various foods with protein sources.

Sources	Number and percentage of students		
	Good Source	Not a good source	Not sure
Poultry	112(91%)	4(3%)	8(6.5%)
Cheese	71(57%)	39(31.7%)	15(12%)
Fruits	46(37%)	66(53.6%)	13(10.5%)
Legumes	89(72%)	17(13.8%)	17(13.8%)
Butter	34(27.6%)	72(58.5%)	18(14.6%)
Nuts	93(75.6%)	25(20.3%)	6(4.8%)

**Table 2:** Depicts the responses related to calcium content of a glass of whole milk compared to skimmed milk.

Calcium content	Frequency (n = 123)	Percentage
About the same	34	27.6%
Much higher	46	37.4%
Much lower	29	23.7%
Not sure	23	18.7%

**Table 3:** Depicts the responses related to predominant type of fat found in various foods given below.

Sources	Number and percentage of students				
	Polyunsaturated	Monounsaturated	Saturated	Cholesterol	Not sure
Olive Oil	45(36.5%)	43(34.9%)	7(5.69%)	4(3.25%)	28(22.7%)
Butter	46(37.6%)	27(21.9%)	19(15.44%)	18(14.6%)	20(16.2%)
Sunflower Oil	52(42.2%)	32(26%)	10(8.13%)	5(4%)	27(21.9%)
Eggs	44(35.7%)	44(35.7%)	3(2.4%)	8(6.5%)	36(29.2%)

**Table 4:** Depicts the responses related to highest amount of trans-fat content of various food as shown below.

Sources	Frequency (n = 123)	Percentage
Biscuit, cakes & pastries	93	75.6%
Rapeseed Oil	12	15.4%
Fish	19	9.8%
Eggs	7	5.7%
Not sure	20	16.3%

**Table 5:** Depicts the response of students for starchy foods sources from the given set of foods

Sources	Number and percentage of students		
	Starchy Food	Not a Starchy Food	Not sure
Cheese	37(30%)	65(52.8%)	24(19.5%)
Potato	108(87%)	8(6.5%)	8(6.5%)
Pasta	86(70%)	20(16.2%)	19(15.44%)
Nuts	23(17%)	80(65%)	22(17.8%)
Plantains	41(33%)	32(26%)	51(41.4%)

**Table 6:** Depicts the responses related to the following nutrients containing most calories for the same weight of food.

Nutrients	Frequency (n = 123)	Percentage
Sugar	41	33.3%
Starchy	29	23.6%
Fiber/roughage	16	13%
Fat	51	41.5%
Not sure	13	10.6%

**Table 7:** Depicts the responses of comparison of processed foods compared to minimally processed foods

Processed foods are	Frequency (n = 123)	Percentage
High in calories	96	78%
Lower in salt	13	10.6%
Higher in fibre	10	8.1%
Not sure	17	13.8%

Section II: questions about individual dietary choices

Five questions related to healthy choices were asked in section II. Question 1 was associated with selecting yoghurt with the least sugar in it. Seventy-eight percent answered it as natural yoghurt, whereas 8.9% were unsure of the sugar content, as shown in Table 8. Table 9 shows the nutritious method for giving flavour to meals, for which 65% answered herbs as the healthiest option, whereas 13% were unsure. When asked about the cooking method needing fat, 36.8% responded as sauteing, 32.5% grilling, 30.1% baking, and 10.6% steaming, as shown in Table 10. The majority of students, 54.5%, answered that broccoli, carrots and tomatoes have the most varieties of vitamins and minerals, and 7.3% were not sure of the same, as shown in Table 11. Traffic signal coding for the fat content was asked in the last question of this section, for which 47.2% of students answered correctly as medium fat and 17% needed clarification, as shown in Table 12.

**Table 8:** Depicts the choice of healthy yogurt by students

Type of Yoghurt	Frequency (n = 123)	Percentage
0% fat cherry yoghurt	12	9.8%
Natural Yoghurt	96	78%
Creamy Yoghurt	9	7.3%
Not sure	11	8.9%

**Table 9:** Foods which provide flavor to meal.

Foods	Frequency (n = 123)	Percentage
Coconut Milk	28	22.8%
Soy Sauce	14	11.4%
Herbs	80	65%
Not sure	16	13%

**Table 10:** Depicts the responses of students regarding cooking method needing fat as substrate for cooking

Cooking method	Frequency (n = 123)	Percentage
Grilling	40	32.5%
Steaming	13	10.6%
Baking	37	30.1%
Sauteing	45	36.8%

**Table 11:** Depicts the responses of students regarding the healthy salads offering most variety of vitamins and antioxidants.

Salad type	Frequency (n = 123)	Percentage
Lettuce, green pepper & cabbage	40	32.5%
Broccoli, Carrot & Tomatoes	67	54.5%
Red Peppers, Tomatoes & lettuce	19	15.4%
Not sure	9	7.3%

**Table 12:** Depicts the responses of the students regarding the traffic light signaling for the fat content of a food

Fat type	Frequency (n = 123)	Percentage
Low fat	17	13.8%
High fat	35	28.5%
Medium fat	58	47.2%
Not sure	21	17.1%

Section III: questions related to expert advice

This section includes seven questions related to advice given by the experts. Table 13 shows the responses about the food groups people should consume according to experts. Eighty-two percent answered that more fruits were needed to be consumed, whereas 3.2% were unsure. Ninety-three percent said food and drinks with added sugars should be consumed less, according to experts, and 4% are still determining. 80.4% answered vegetables should be eaten more, and 4.8% were unsure. For fatty foods, processed red meat, whole grains and salty food, the majority responded correctly as eaten to less. As shown in Table 14, servings of fruits consumed should be two or more, as answered by 36.6%, whereas only 7.3% responded correctly to five or more servings. Fifty-six percent of students said trans-fat consumption should be less, whereas 20%, 25% and 28% of students are unsure about the unsaturated, saturated and trans-fat consumption, respectively, as shown in Table 15. Table 16 shows the dairy recommendations wherein 60% of students say experts advise consuming reduced fat in skimmed milk, and 21% still need to be sure. The recommendation of oily fish per week is one to two times/week, and 57% of the students and 25% still determine what the recommendations say, as shown in Table 17. The amount of alcoholic drinks advised by experts is no drink per 41.5% of students, whereas 22.8% are not sure yet, as shown in Table 18. Table 19 shows that 37% of students think ¼ plates should be filled with starchy foods according to Eatwell plate, whereas 28% are unsure.

**Table 13:** Depicts the responses related to experts advice about the consumption of various food groups

Food Groups	Number and percentage of student responses			
	More	Same	Less	Not sure
Fruit	101(82%)	19(15.4%)	1(0.8%)	4(3.2%)
Food and drinks with added sugars	12(9.7%)	13(10.5%)	93(75%)	5(4%)
Vegetables	99(80.4%)	13(10.5%)	5(4%)	6(4.8%)
Fatty foods	9(7.3%)	26(21%)	84(68%)	5(4%)
Processed red meat	11(8.9%)	35(28.4%)	64(52%)	16(13%)
Wholegrains	69(56%)	39(31%)	8(6.5%)	9(7.3%)
Salty food	7(5.6%)	25(20%)	81(65.8%)	10(8.1%)
Water	102(82.9%)	9(7.3%)	8(6.5%)	5(4%)

**Table 14:** Depicts the responses related to expert advice about servings of fruits and vegetables consumption/person/day.

Servings	Frequency (n = 123)	Percentage
Two	45	36.6%
Three	36	29.3%
Four	23	18.7%
Five or more	9	7.3%
Not sure	13	10.6%

**Table 15:** Depicts the responses related to kind of fat that specialist advised against eating too much of.

Sources	Percentage of student responses		
	Eat less	Not eat less	Not sure
Unsaturated fats	36%	43%	20%
Saturated fats	47%	25%	25%
Trans fats	56%	13%	28%

**Table 16:** Depicts the responses related to type of dairy recommended by experts to drink.

Sources	Frequency (n = 123)	Percentage
Full fat	18	14%
Reduced fat(skimmed & semi – skimmed milk)	60	48%
Mixture of full & reduced fat	25	20%
Neither, dairy food should be avoided	10	8.1%
Not sure	21	17%

**Table 17:** Depicts the responses related to consumption of oily fish like (salmon & mackerel) by experts

Frequency of fish consumption	Frequency (n = 123)	Percentage
1-2 times/week	71	57%
3-4 times/week	18	14%
Every day	6	5%
Not sure	31	25%

**Table 18:** Depicts the responses related to amount of alcoholic drinks advised by experts per day

Amount of alcoholic drink	Frequency (n = 123)	Percentage
1 drink each for men & women	9	7.3%
2 drinks each for men & women	14	11.4%
2 drinks for men & 1 drink for women	16	13%
3 drinks for men & 2 drinks for women	9	7.3%
No drink is recommended	51	41.5%
Not sure	28	22.8%

**Table 19:** Depicts the responses related to proportion of starchy food in the diet according to ‘Eatwell Plate’

Quantity	Frequency (n = 123)	Percentage
1/4 plate	46	37%
1/3 plate	31	25%
1/2 plate	18	14%
Not sure	35	28%

Section IV: questions related to awareness of health problems

Section 4 was related to awareness regarding health problems. It consisted of a total of 13 questions in it. Seventy-four percent of students, as shown in Table 20, answered correctly regarding the diseases related to low fibre consumption as bowel disorders, whereas 10% were unsure. For diseases related to high sugar consumption, as shown in Table 21, 61.5% answered tooth decay; surprisingly, 25.4% answered hypertension, and 15.6% answered not sure. For diseases related to high salt consumption, 65.9% responded correctly to high blood pressure, whereas 33.3% answered as hypothyroidism, 12% as diabetes, and 10% as not sure, as shown in Table 22.

**Table 20:** Depicts the responses of students indicating the diseases associated with low fibre consumption

Diseases	Frequency (n = 123)	Percentage
Bowel disorders	91	74%
Anaemia	13	10%
Tooth decay	12	9.8%
Not sure	10	8.1%

**Table 21:** Depicts the responses of students indicating the diseases related to high sugar consumption

Diseases	Frequency (n = 123)	Percentage
High Blood Pressure	31	25.4%
Tooth decay	75	61.5%
Anaemia	13	10.7%
Not sure	19	15.6%

**Table 22:** Depicts the responses of students indicating the diseases related to high salt consumption

Diseases	Frequency (n = 123)	Percentage
Hypothyroidism	41	33.3%
Diabetes	12	9.8%
High Blood Pressure	81	65.9%
Not sure	10	8%

Regarding food consumption for cancer reduction, 69.9% answered that abstinence from additives would reduce cancer risk, 25% said avoiding red meat would reduce it, and 10.6% were not sure of the same. For the prevention of heart diseases, 58.5% said consumption of trans fats should be avoided, whereas 17% of students were unaware. For the prevention of diabetes, 53.7% of students said eating less refined food is advisable, and 66.7% of students said animal fats are likely to increase blood cholesterol levels. The knowledge about the glycaemic index of the foods was tested by asking about various food groups, for which 54.5% answered fruits and vegetables have a low glycaemic index, whereas 22.8% were not sure.

The next set of questions was on weight management; for maintenance of healthy weight, 69.9% of students said a complete cut off of fat is not necessary for healthy weight management, 21.1% said it is needed, and 11.4% were not sure. Similarly, that eating bread increases weight was disagreed by 45.5%, agreed by 37.4% and not sure by 18.7%. High protein consumption and maintaining weight were answered by 64.2% of students.

For reducing weight, 53.7% said fibre intake helps to reduce weight, whereas 21.1% were not sure. When asked which body shape increases cardiovascular disease risk, 61% answered it correctly as apple shape, 26% as pear shape and 16.3% as not sure.

## Discussion

This study assessed the medical students’ knowledge and attitude about nutrition and nutrition-related diseases. The gender distribution of participants in this study was 57.3% (66) female and 47.7% (50) male. Participants in the study were 18.56 ± 1.16 years old on average. In a similar research by Alissa et al. of 200 medical students, women comprised 68%, while men comprised 32% [14]. In contrast to our study, research by Alghamdi et al. on 386 students showed (310) 80.3% male and (76) 19.7% female with an average age of 21.5 ± 2.10 years [15]. Similarly, a study by Kumar et al. on 200 subjects showed that 62% of the subjects were males and 38% were females [16].

Knowledge about nutrition

It was discovered in the current study that students’ general understanding of a nutritious diet was sound. 39.8% of students answered the precise requirement of carbohydrates (45 - 65%), fat (10 -35%) and protein (20 -35%), and 43.9% of students said 20 - 30 grams of fibre as recommended. The present study’s findings align with another survey by Alissa et al., which stated that 75-94% of the participants understood what constitutes a balanced diet and how crucial it is to have adequate vitamins and minerals [14].

Although the majority of participants (Table 1) were aware of the foods that are rich in added sugars (Figure 1), salts (Figure 2), and fibre (Figure 3) content, still a chunk of students (8% - 27%) were not sure of the sugar, salt and fibre content. They were not able to make healthy choices. Thirteen percent to 23% of students weren’t sure about the added salt in foods like breakfast cereals, frozen vegetables, bread, red meat and crisps, and 43% reported that bread has low salt. Similar to our study, another study’s results demonstrated that most students knew very little about the primary foods that add salt to the diet, such as cornflakes, pita bread, and Iranian bread, which are foods rich in added salt [17]. In addition, 21.1% of students were unsure of added sugars in foods like diet cola, natural yoghurt, ice cream, tomato ketchup and melon. 17.8% and 11.3% think natural yoghurt and melon are high in added sugar, respectively, whilst 8.1% and 23.5% think ice cream and tomato ketchup are low in added sugar, respectively.

Additionally, 7.3% to 24.8% of students are unaware of the fibre content of foods like oats, bananas, white rice, eggs, potato skin and whole wheat, which falls in line with another study by Schapira et al., wherein 90% of students are unaware of the fibre content of the given food [18]. A large number of students correctly pointed out the foods with high protein sources, were able to identify the starchy foods, and found the predominant fat from the given sources. When asked about foods containing the highest amount of trans fat, 75% recognised it correctly. In addition, students had limited knowledge about the calcium content of skimmed milk and calorie content per weight of the nutrients. This ignorance may help to explain the results of earlier research showing that college students consume low amounts of dietary fibre [19] and large quantities of salt and fat [20,21].

Individual dietary choices

Students were enquired about their individual dietary choices (Table 2). Seventy-eight percent of students chose the correct answer for the least sugar content of the yoghurt, and 61% of students were aware of the low-fat content of the soup. Sixty-five percent showed awareness about the healthy alternatives of adding extra flavour to food in the form of herbs, and there was a mixed response from students when asked about the cooking method requiring less fat, with 45% answering it as sauteing, 32.5% as grilling, 30% as baking and 10% as steaming. Vegetables containing the most variety of vitamins and antioxidants were picked out correctly by 54.5% of students, and 7.3% needed clarification. Students displayed mixed responses for questions related to traffic light colour coding for foods; when asked about the meaning of amber colour, 58% answered as medium fat content correctly, 35% answered as high-fat content, 17% as low fat and 21% as unsure. Overall, students displayed adequate knowledge about healthy food options but lacked awareness about the healthy cooking methodology and colour coding system. A study by Alhazmi et al., similar to the present study, demonstrated that the majority of medical students were well acquainted with healthy choices [22]; on the contrary, according to Sam et al., eating habits among college students frequently don’t match what they know [23].

Knowledge about expert’s advice

As shown in Table 3, 36.6% of students think that experts advise people to eat two servings of fruits daily, 7.3% think they advise eating five or more servings, and 10.6% still need to decide. The majority (41.5%) of students believe experts recommend zero drinks of alcohol per day, and 22.8% need to figure out what advice is being given concerning alcohol. Thirty-seven percent of students are aware, and 28% are unaware of the starchy food content of the ‘Eatwell plate’ guideline. Fifty-seven percent of students think experts advise eating one to two times/week of fish (salmon and mackerel) per week, whereas 25% are unsure. Similarly, 56% said that experts advised patients not to eat trans fat too much, while 28% are unsure. In addition, 48% said skimmed/semi-skimmed milk should be consumed, while 17% were uncertain regarding the milk advice. The lack of proper knowledge and awareness about the expert’s advice noticed in the present study is attributed to the scarce nutrition curriculum in medical schools. Similar to the present study, a survey by Macaninch et al. also showed that medical students felt that nutrition education was inadequate [24].

Awareness about the health problems or diseases related to diet and weight management

Awareness about the diseases related to diet, weight management and health problems is tabulated in Table 4. Seventy-four percent of students answered that bowel diseases are related to low intake of fibre and 8.1% are not sure. When asked about diseases related to consumption of sugars, 61.5% answered tooth decay, and 15.6% were unsure. When asked about diseases related to salt consumption, 65.9% answered about high blood pressure, 33.3% about hypothyroidism, 9.8% about diabetes, and 8% were unsure. 69.9% said avoiding additives in food will reduce the chances of getting cancer, while 10.6% said drinking alcohol regularly reduces the chances of cancer. 58.5% of students thought eating less trans fats prevents heart diseases, while 17% were unsure how to prevent heart diseases. Sixty-one percent of students correctly pointed out that an apple-shaped body increases the risk of cardiovascular diseases, whereas 26% still think a pear-shaped body leads to cardiovascular diseases, and 16.3% are still unsure. 66.7% said animal fats raise blood cholesterol, and 54.5% answered that white bread increases blood sugars rapidly. It can be noted that most of the students are aware of the diet-disease relationship in the current study, similar to another study whose students had good knowledge about diet-related diseases [13].

69.9% of students disagreed with the statement that people should completely cut fat from their diet to maintain a healthy weight. 64.2% agreed that one should eat a high-protein diet to maintain a healthy diet. Similarly, 45.5% disagreed with the statement that eating bread always causes weight, while 53.7% agreed that fibre intake decreases the chances of weight gain. The current study’s findings align with the survey by Belogianni et al., which states that some students responded that conventional approaches include eating a high-protein diet, taking nutritional supplements, and limiting fat in the diet to maintain a healthy diet [13].

Overall, students’ knowledge of the four sections is inadequate, and they are less confident about the counselling to be done and treatment to be provided in terms of diet and nutrition for their patients due to their lack of knowledge about diet-related diseases. As per the 2019 Lancet systemic review on “Nutrition in medical education,” half of the medical students scored lower than the school’s passing rate on tests designed to evaluate nutrition knowledge, and only half of the newly graduated doctors correctly answered the questions. According to other studies, there needs to be more understanding of nutrition among medical students. Furthermore, there is a need for further debate regarding the minimal level of nutrition knowledge that medical students should possess, which complicates determining how appropriate this knowledge is. As a result, a universal standard for university education should be set for the necessary degree of nutrition knowledge. The lack of understanding of nutrition persists in medical practice, with medical students feeling ill-prepared to handle circumstances where definitive nutrition therapy has the most significant potential to impact patient outcomes. It is logical to believe that doctors cannot give patients the best care possible if they are not adequately educated about nutrition. The EAT-Lancet Commission framework’s idea of the right to health, which addresses social justice issues, food insecurity, water scarcity, climate change, preventive health care, and access to healthcare, is pertinent to this deficiency [25].

Implications

Nutrition is a critical component in the prevention and treatment of chronic diseases such as obesity, metabolic syndrome, diabetes, etc. Nutritional guidance can assist aspiring medical professionals in caring for themselves, their families, and their patients. Medical education must be improved by an institutional commitment to make nutrition education mandatory in training, nutrition competencies to provide a benchmark for nutrition knowledge and skills to be included in curricula, and funding to research and develop innovative curriculum initiatives to guarantee graduating medical students are supported throughout their education to provide optimal nutrition care to patients. With these advancements in medical nutrition education, physicians will be more equipped to encourage healthy eating and lifestyle choices among their patients and the general public [25].

Strengths

To the best of our knowledge, it is the first nutritional knowledge assessment study conducted using medical students in our country based on a general nutrition knowledge questionnaire. 

Limitations

One of the study’s drawbacks is the homogeneity of the study sample from a single medical college, the results of which cannot be generalised. Another is inadequate sample size and representativeness due to a significant dropout rate.

## Conclusions

Student’s response reveals that relatively few of them are aware of the fat, salt, and fibre content, pointing to gaps in their understanding of these nutrients and the foods that contain them. Students showed a sufficient level of understanding of the healthy food selections, but they were not aware of the cooking methods. Students are not confident about experts’ advice related to disease prevention, but they are aware of the diet-disease relationship. The current study’s findings about students’ inadequate understanding and nutrition awareness are explained by the limited nutrition curricula in medical schools. Education-based approaches through nutrition experts should be inculcated in the medical curriculum to fill the knowledge gaps among medical students, which will help them to adopt a holistic approach towards patient health.
